# Multidimensional Evaluation of All-Cause Mortality Risk and Survival Analysis for Hospitalized Patients with COVID-19

**DOI:** 10.7150/ijms.58889

**Published:** 2021-06-26

**Authors:** Jingwen Li, Hu Luo, Gang Deng, Jinying Chang, Xiaoming Qiu, Chen Liu, Bo Qin

**Affiliations:** 1 Department of Infectious Diseases, the First Affiliated Hospital of Chongqing Medical University, Chongqing, China.; 2 No 1. Intensive Care Unit, Huoshenshan Hospital, Wuhan, China.; 3 Department of Respiratory and Critical Care Medicine, Southwest Hospital, Third Military Medical University (Army Medical University), Chongqing, 400038, China.; 4 Department of Radiology, Guanggu district, Hubei Maternal and Child Health Hospital, Wuhan, China.; 5 63650 Hospital of PLA, Malan, 841700, China.; 6 College of Mathematics and Statistics, Chongqing University, Chongqing, China.; 7 Department of Radiology, Huangshi Central Hospital, Affiliated Hospital of Hubei Polytechnic University, Huangshi, China.; 8 Department of Radiology, Southwest Hospital, Third Military Medical University (Army Medical University), Chongqing, 400038, China.

**Keywords:** COVID-19, mortality risk, survival analysis

## Abstract

**Background:** Coronavirus disease 2019 (COVID-19) has caused over 3.8 million deaths globally. Up to date, the number of death in 2021 is more than that in 2020 globally. Here, we aimed to compare clinical characteristics of deceased patients and recovered patients, and analyze the risk factors of death to help reduce mortality of COVID-19.

**Methods:** In this retrospective study, a total of 2719 COVID-19 patients were enrolled, including 109 deceased patients and 2610 recovered patients. Medical records of all patients were collected between February 4, 2020, and April 7, 2020. Clinical characteristics, laboratory indices, treatments, and deep-learning system- assessed lung lesion volumes were analyzed. The effect of different medications on survival time of fatal cases was also investigated.

**Results:** The deceased patients were older (73 years versus 60 years) and had a male predominance. Nausea (10.1% versus 4.1%) and dyspnea (54.1% versus 39.2%) were more common in deceased patients. The proportion of patients with comorbidities in deceased patients was significantly higher than those in recovered patients. The median times from hospital admission to outcome in deceased patients and recovered patients were 9 days and 13 days, respectively. Patients with severe or critical COVID-19 were more frequent in deceased group. Leukocytosis (11.35×10^9^/L versus 5.60×10^9^/L) and lymphocytopenia (0.52×10^9^/L versus 1.58×10^9^/L) were shown in patients who died. The level of prothrombin time, activated partial prothrombin time, D-dimer, aspartate aminotransferase, alanine aminotransferase, urea, creatinine, creatine kinase, glucose, brain natriuretic peptide, and inflammatory indicators were significantly higher in deceased patients than in recovered patients. The volumes of ground-glass, consolidation, total lesions and total lung in all patients were quantified. Complications were more common in deceased patients than in recovered patients; respiratory failure (57.8%), septic shock (36.7%), and acute respiratory distress syndrome (26.6%) were the most common complications in patients who died. Many treatments were more frequent in deceased patients, such as antibiotic therapy (88.1% versus 53.7%), glucocorticoid treatment (70.6% versus 11.0%), intravenous immunoglobin treatment (36.6% versus 4.9%), invasive mechanical ventilation (62.3% versus 3.8%). Antivirals, antibiotics, traditional Chinese medicines and glucocorticoid treatment may significantly increase the survival time of fatal cases. Quantitative computed tomography imaging results were correlated with biochemical markers.

**Conclusions:** Most patients with fatal outcomes were more likely to have common comorbidities. The leading causes of death were respiratory failure and multiple organ dysfunction syndrome. Acute respiratory distress syndrome, respiratory failure and septic shock were the most common serious complications. Antivirals, antibiotics, traditional Chinese medicines, and glucocorticoid treatment may prolong the survival time of deceased patients with COVID-19.

## Introduction

To date, more than 176 million cases of coronavirus disease 2019 (COVID-19) have been diagnosed and over 3.8 million patients died from this infectious disease globally. In early 2021, World Health Organization (WHO) confirmed that the incidence rate of COVID-19 reached a peak again 1. COVID-19 is highly contagious, the value of the basic reproduction ratio (R0) is up to 5.7 2. Several severe acute respiratory syndrome coronavirus 2 (SARS-CoV-2) variant strains have increased the difficulty in controlling viral transmission and therapeutic intervention 3, 4. Although several SARS-CoV-2 vaccine candidates are currently undergoing clinical trials, evidence of their effectiveness and safety needs to be confirmed 5, 6. Reducing new COVID-19 infections may be the most effective method for epidemic prevention and control.

The majority of COVID-19 patients present mild to moderate clinical symptoms, and severe cases are more likely to develop acute respiratory distress syndrome (ARDS), acute myocardial injury and acute liver dysfunction; kidney injury can also occur 7. The virological characteristics and specific pathogenic mechanisms of SARS-CoV-2 remain to be elucidated. In China, therapeutic medications for COVID-19 mainly include antivirals, antibiotics, glucocorticoids, convalescent plasma, hyperimmune immunoglobulins, immunomodulatory therapy, and traditional Chinese medicines 8, 9. However, there is no conclusive evidence regarding the use of these medications 10-12.

In this study, we aimed to report the clinical characteristics of 109 deceased patients and 2610 recovered patients with COVID-19, evaluate the impact of different medications on the survival time of patients who died, analyze the correlation between quantitative computed tomography (CT) values and clinical parameters. We hope that this study will provide useful information for early identification of patients with high-risk of death and medical treatment selection.

## Materials and methods

### Research design and participants

This multicenter retrospective study enrolled 109 deceased patients and 2610 recovered patients with COVID-19. The clinical features of all patients and the effects of different medications on the survival time of fatal patients were investigated. All patients were recruited from three hospitals in Hubei: Huoshenshan Hospital, Hubei Maternal and Child Health Hospital (Guanggu District), Huangshi Central Hospital. All patients were hospitalized between February 4, 2020 and April 7, 2020. The patients enrolled in this study were diagnosed according to the Diagnosis and Treatment Protocol for COVID-19 by the National Health Commission (NHC) of China (trial seventh edition); the disease severity of COVID-19 was classified as mild, moderate, severe, or critical according to the same protocol of China NHC 13. The inclusion criteria were patients diagnosed with COVID-19 who had fatal outcomes. The exclusion criteria were as follows: 1) patients aged <18 years; 2) patients without clinical and laboratory data. A total of 109 patients were enrolled in this study.

### Data collection

Medical information of all patients was extracted from the electronic medical records, including epidemiological data, clinical characteristics, laboratory tests, CT imaging data and treatment data. All data were reviewed by two investigators (H. L. and G.D.) and entered into the computer database by two analysts (JW. L. and JY.C.) independently. The primary outcome was all-cause death during hospitalization.

### CT quantitative analysis

**Figure 1:**
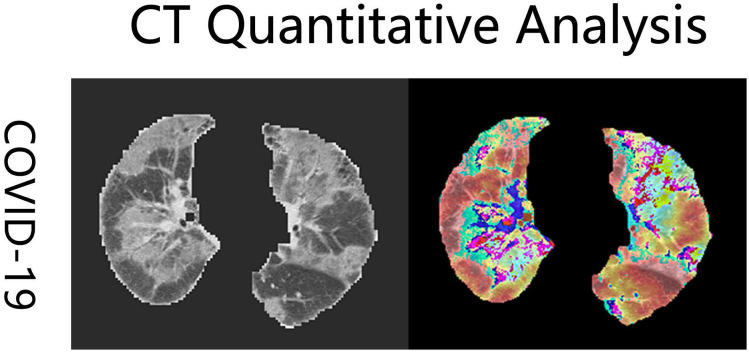
Examples of coronavirus disease 2019 (COVID-19) computed tomography (CT) imaging heatmaps created from feature maps with a deep learning model and imposed on the raw CT imaging. The heatmaps are a standard Jet colormap, warm colors highlight the activation region associated with the predicted class.

All patients underwent a standard chest CT scans from the above-mentioned three hospitals. CT scans were performed using a 64-row spiral CT scanner (Berlin, Germany). The first chest CT examination after hospital admission was extracted and analyzed 14. All imaging datasets were anonymized. Each scan had a different slice thickness from 0.5 mm to 3 mm. To reduce the discrepancies arising from spatial resolution among the scans, the CT images were interpolated to 1 mm ×1 mm ×1 mm in each scan and all CT numbers (Hounsfield units) were normalized with the lung window center, -500 HU/lung window width, 1,500 HU. Pneumonia lesion volumes were quantified using an in-house deep learning computational algorithm (Fig. 1) 15. The details of this deep learning model have been described in our previous articles 15, 16. Three functional modules were included in this model: lung segmentation, pneumonia lesion segmentation, and quantitative analysis. Total lung volumes, ground-glass opacity volumes, consolidation volumes, whole lung pneumonia volumes, and the lesion ratio (total lesion volumes/total lung volumes) were calculated.

### Statistical analysis

Categorical variables were expressed as counts and percentages. Continuous variables were expressed as the mean ± standard deviation or as medians and interquartile ranges (IQRs). Categorical data were compared using χ^2^ test or Fisher's exact test. Continuous variables were compared using the *t*-test or Mann-Whitney U test. Survival curves were estimated using the Kaplan-Meier method. A two-sided P-value <0.05 was considered statistically significant. We computed the correlations between the clinical parameters and the CT imaging analysis results using Pearson's correlation coefficient. Data were analyzed using SPSS Statistics software (version 25.0, SPSS Inc., College Station, TX, USA).

This study was conducted in accordance with the Declaration of Helsinki and was approved by the Institutional Ethics Board of the First Affiliated Hospital of Army Medical University Ethics Committee (Approval No. KY2020036). The requirement for written informed consent was waived according to the policy of emerging infectious diseases issued by the NHC of the People's Republic of China.

## Results

### Demographic and clinical characteristics

Totally, 109 deceased patients and 2610 recovered patients were examined in this study. For all patients, the median age was 61.0 (IQR, 50.0-68.0) years and 1368 (50.2%) patients were men. The most common symptoms were fever (1993 patients [73.3%]), cough (1992 patients [73.3%]), anorexia (1644 patients [60.5%]) and fatigue (1541 patients [56.7%]). The most common comorbidities were hypertension (859 patients [31.6%]), diabetes (399 patients [14.7%]), and cardiovascular disease (211 patients [7.8%]). The median length from hospital admission to outcome was 13.0 days (IQR 8.0-19.0) and the median duration from symptoms onset to outcome was 27.0 days (IQR 19.0-37.0). For disease severity status in all patients, 1915 (70.4%) patients were mild or moderate and 804 (29.6%) patients were severe or critical (Table 1).

Compared with recovered patients, deceased patients were older (73.0 years versus 60.0 years) and male patients were in the majority (65.1% versus 49.6%). Nausea (10.1% versus 4.1%) and dyspnea (54.1% versus 39.2%) were more commonly exhibited by deceased patients. 1301 (49.8%) recovered patients and 101 (92.7%) deceased patients had at least one chronic medical condition. The proportion of patients with all listed comorbidities in deceased patients was significantly higher than those in recovered patients. The median times from hospital admission to outcome (9.0 days versus 13.0 days) and the median times from symptom onset to outcome (23.5 days versus 27.0 days) were shorter in deceased patients. Furthermore, the percentages of patients with severe or critical COVID-19 were significantly higher in deceased group than in recovered group (97.2% versus 26.7%). The leading cause of death with COVID-19 was respiratory failure (57 [52.3%]); other causes of death included multiple organ dysfunction syndrome (MODS) (36 [33.3%]), septic shock (33 [30.3%]), and sudden cardiac death (9 [8.3%]) (Table 1).

### Laboratory results and radiologic findings

Numerous differences in laboratory findings between the two groups were found (Table 2). Compared with recovered patients, deceased patients had higher levels of white blood cells (11.35×10^9^/L versus 5.6×10^9^/L) and neutrophils (10.22×10^9^/L versus 3.15×10^9^/L); the levels of lymphocytes (0.52×10^9^/L versus 1.58×10^9^/L) and platelets (132.0×10^9^/L versus 224.0×10^9^/L) were lower deceased patients. Prothrombin time (15.5s versus 12.0s), activated partial prothrombin time (33.6s versus 31.1s) and D-dimer (5.35 mg/L versus 0.98 mg/L) in deceased patients were increased. Aspartate aminotransferase, alanine aminotransferase, urea, creatinine, creatine kinase, glucose and brain natriuretic peptide (BNP) in deceased patients were all higher than in recovered patients. The level of albumin (29.45 g/L versus 38.50 g/L) in deceased patients was lower. C-reactive protein (CRP) (107.02 mg/L versus 5.84 mg/L), procalcitonin (PCT) (0.85 ng/mL versus 0.05 ng/mL) and interleukin-6 (303.01 pg/mL versus 10.35 pg/mL) in deceased patients were significantly higher than in recovered patients.

Pneumonia lesions in deceased and recovered group were detected and analyzed. As shown in Table 3, the ground-glass opacity (GGO) volumes, total lesion volumes and the lesion ratio (total lesion volumes to total lung volumes) were significantly larger in deceased patients than in recovered patients. Consolidation volumes and total lung volumes had no significant differences between the two groups.

### Complications and treatments

As shown in Table 4, respiratory failure (57.8%), acute respiratory distress syndrome** (**ARDS) (26.6%), and septic shock (36.7%) were the most common complications in COVID-19 patients who died. Furthermore, the proportion of patients with complications in deceased patients was significantly higher than that in recovered patients, including respiratory failure (57.8% versus 2.6%), ARDS (26.6% versus 1.3%), acute cardiac injury (15.6% versus 2.8%), acute kidney injury (10.1% versus 3.3%), and septic shock (36.7% versus 1.2%).

The main treatments for COVID-19 patients in this study are presented in Table 4. Antibiotic treatment mainly included piperacillin, moxifloxacin, and cephalosporins. Antiviral treatment mainly included ribavirin, arbidol, oseltamivir, and ritonavir. Traditional Chinese medicines included Lian-Hua Qing-Wen Granule (LHQWG), Xue-Bi-Jing (XBJ), and Jin-Hua Qing-Gan Granules (JHQGG). These Chinese patent medicines have different antiviral effects; LHQWG was mainly for influenza and immune regulatory 17; XBJ was mainly for severe infection such as sepsis and systemic inflammatory response syndrome 18; JHQGG was mainly for influenza with fever, weakness, cough, headache and sore throat 19. The targeted immunomodulatory therapy included tocilizumab (an inhibitor of IL-6). Antibiotic treatment (55.1%), antiviral treatment (63.3%), traditional Chinese medicines treatment (79.8%) and oxygen therapy (70.0%) were the most common treatment for COVID-19 patients. Compared with recovered patients, many treatments were more frequent in deceased patients, including antibiotic therapy (88.1% versus 53.7%), glucocorticoid treatment (70.6% versus 11.0%). intravenous immunoglobulin treatment (36.6% versus 4.9%), targeted immunomodulatory treatment (15.6% versus 3.3%), non-invasive mechanical ventilation (30.3% versus 8.0%), invasive mechanical ventilation (62.3% versus 3.8%) and continuous renal replacement therapy (CRRT) (11.9% versus 0.7%), extracorporeal membrane oxygenation (ECMO) (1.8% versus 0.0%). Antiviral treatment, traditional Chinese medicines treatment, convalescent plasma treatment and oxygen therapy showed no significant differences in the two groups.

### Survival analysis

**Figure 2:**
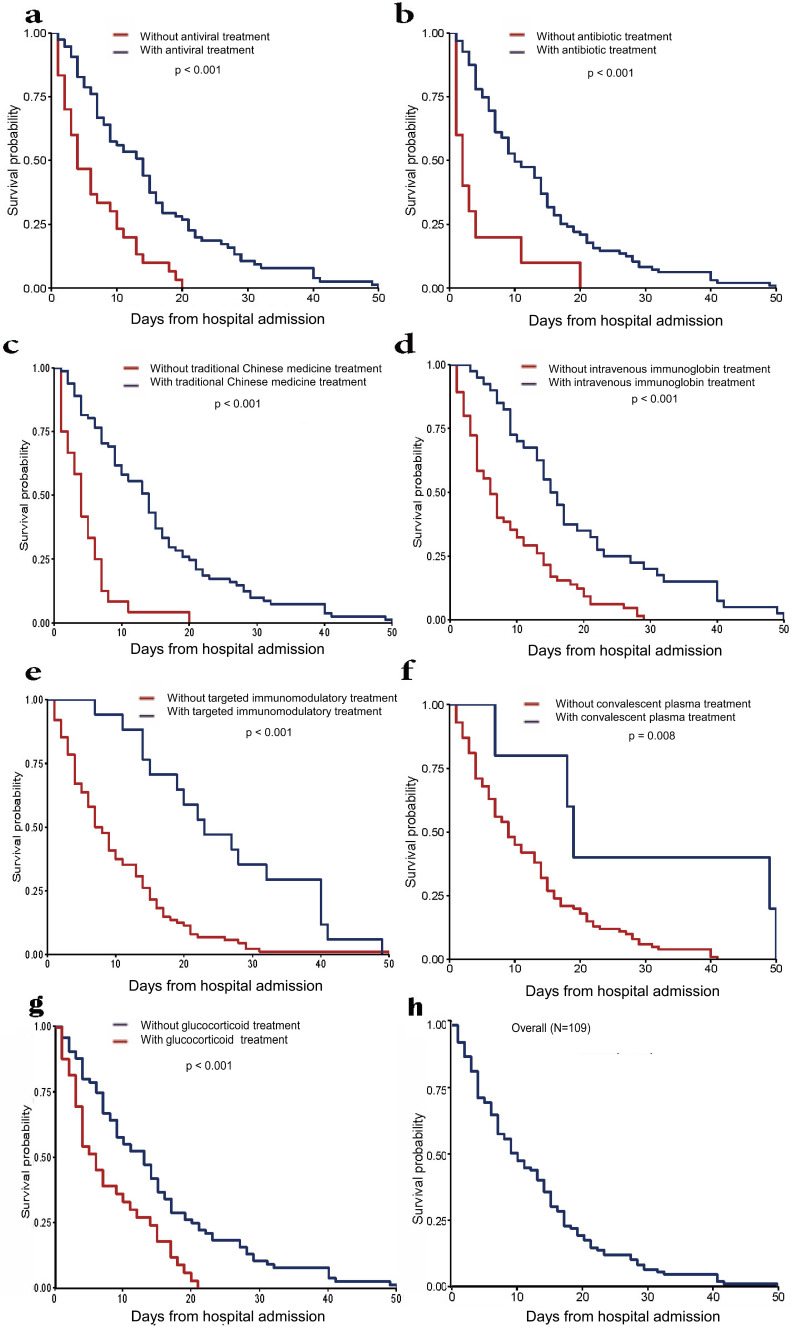
Survival time from hospital admission to death in deceased patients. (a) Patients treated with antibiotics treatment *vs*. patients treated without antibiotics treatment. (b) Patients treated with antiviral treatment *vs*. patients treated without antiviral treatment. (c) Patients treated with traditional Chinese medicines treatment *vs*. patients treated without traditional Chinese medicines treatment. (d) Patients treated with intravenous immunoglobin treatment *vs.* patients treated without intravenous immunoglobin treatment. (e) Patients treated with targeted immunomodulatory treatment *vs*. patients treated without targeted immunomodulatory treatment. (f) Patients treated with convalescent plasma treatment *vs*. patients treated without convalescent plasma treatment. (g) Patients treated with glucocorticoid treatment *vs.* patients treated without glucocorticoid treatment. (h) Overall survival time of all patients.

We further analyzed the survival time of fatal cases from hospital admission to death (Fig. 2). For deceased patients, the median length from hospital admission to death was 9.0 days (IQR 4.0-17.0) (Table 1). Antivirals, antibiotics, traditional Chinese medicines and glucocorticoid treatment may significantly prolong the survival time compared with the effect of intravenous immunoglobulin therapy. COVID-19 convalescent plasma therapy showed a relatively weak effect. Tocilizumab showed the weakest effect on survival time in this study.

### Correlation analyses

Pearson's correlation was performed between CT quantitative values and clinical parameters in all COVID-19 patients (Table 5). For deceased patients, CRP levels (*p* < 0.05) were found to have positive correlations with all CT quantitative values; PCT level, neutrophils counts and D-dimer were positively correlated with the consolidation volumes (*p* < 0.05); the lesion ratio showed positive correlation with D-dimer level (*p* < 0.01). For recovered patients, CRP level and neutrophil counts were found to have positive correlations with all CT quantitative values (*p* < 0.05). No significant differences were found in other indicators.

## Discussion

This study included COVID-19 patients with fatal outcomes. We described the clinical characteristics of these patients, quantified lung lesions using an artificial intelligence method, and analyzed the effects of different medicine therapies on survival time from hospital admission to death. Although the majority of COVID-19 cases are mild to moderate in severity, as of April 2021, over 3.0 million patients died due to the highly contagious nature of SARS-CoV-2 1. The rapidly increasing number of patients causes a considerable burden on medical staff, and the clinical value of fatal cases is easily ignored due to inadequate manpower and limited time 20. We hope that this study will help identify patients at high risk of death earlier by recognizing some characteristic clinical features and provide valuable information for effective treatments.

In accordance with recent reports, the majority of patients were men, and the most common clinical characteristics of patients included fever, cough, dyspnea, and fatigue 21, 22. These indicators suggested that patients with multiple symptoms on admission, such as those mentioned above, may be in severe condition. Comorbidities including hypertension, diabetes, and cardiovascular disease have been proven to be high-risk factors of death, and these diseases interactively promote the pathological progression of COVID-19 23, 24. Patients with comorbidities should be monitored intensively, glycemia and blood pressure control are crucial in decreasing the mortality of COVID-19.

The abnormal laboratory indices were similar to those reported in previous studies 21, 25. Most patients had leukocytosis, neutrophilia, and increased procalcitonin levels, which suggested that a large proportion of fatal cases may develop severe bacterial infections. Severe lymphopenia is a characteristic change in COVID-19 patients, and some research studies have reported that this is caused by the direct attack of SARS-CoV-2 on lymphocytes 26, however, the potential mechanisms still need to be defined. Coagulation disorders (e.g., prolonged prothrombin time and D-dimer elevation) are also common in patients, and D-dimer elevation is another important predictor of poor prognosis 22. The exact mechanisms are unclear, and whether SARS-CoV-2 can directly damage vascular endothelial cells needs to be verified 27. Other organ dysfunctions were observed, including liver, kidney, and myocardial injury characterized by abnormal damage-specific enzymes. These changes indicated that COVID-19 was associated with progressive systemic damage.

In addition to respiratory failure and multiple organ dysfunction, septic shock is one of the most common causes of death in COVID-19 patients 21, 28. Some studies have shown that evidence of bacterial infections was not found in patients on admission; thus, SARS-CoV-2 infection might be the direct cause of septic shock. Meanwhile, previous study has shown that viral infection can cause sepsis in nearly 40% of adults with community-acquired pneumonia 22, 26, 29. It was speculated that SARS-CoV-2 may attack lung capillary endothelial cells and other organs directly; systemic inflammatory response, immunosuppression, and microcirculation dysfunction together lead to viral sepsis 9, 30. However, consistent with previous reports 21, 25, the white blood cell and neutrophil counts were elevated in more than half of the fatal cases of COVID-19 in this study, and the procalcitonin level was increased. These abnormalities suggested that COVID-19 patients may develop secondary bacterial infections. Survival time analysis showed that antibiotic therapy might prolong survival time, which supported bacterial infections. Previous studies have described the secondary bacterial infections in COVID-19 patients 31, 32. According to Surviving Sepsis Campaign guidelines on the management of critically ill adults with COVID-19, empiric antibiotic therapy was recommended to use in patients with respiratory failure and mechanical ventilation 33. Further prospective studies are required for appropriate stewardship interventions of COVID-19 with bacterial infections.

Moreover, we analyzed the effects of different treatments on survival time from hospital admission to death. The effect of antivirals and antibiotics on increasing the survival time was obvious, indicating that strong anti-infective therapy was the most effective treatment throughout the course of COVID-19. Although COVID-19 convalescent plasma therapy in this study was effective, this therapy is being debated; side effects such as severe transfusion-associated dyspnea, transfusion-related acute lung injury, and allergic transfusion reaction, have been reported 34, 35. More evidence is required to validate its efficacy and safety. Tocilizumab had the weakest effect on the survival time of patients, which is different from the results of recent studies. We speculated that this may be due to the small sample size 36, 37. Traditional Chinese medicines, as endemic medicines in China, also had a good impact on the survival time in this study. Previous studies have reported the mechanisms of action of these different traditional Chinese medicines. Zhong et al. found that LHQWG could repress SARS-COV-2 replication obviously, affect virus morphology and exhibit anti-inflammatory activity *in vitro* 38. In acute lung injury mouse model, LHQWG acted as a potent epithelial protector, significantly reducing NF-κB levels, reversing the SOCS3 expression in macrophages, and blocking proapoptotic communication between macrophages and alveolar epithelial cells 39. XBJ has been reported to significantly prevent cell death by blocking SARS-CoV-2 proliferation and inhibiting the expression of many pro-inflammatory cytokines expressions such as IL-6, IFN-γ, TNF-α and IL-10 40-42. JHQGG could regulate multiple signaling pathways via binding to ACE2, it also reduces host inflammation and activates antiviral immunity by inhibiting virus replication and binding to target cells 43, 44.

Glucocorticoid treatment could increase the survival time of patients, who died of COVID-19. During the early stages of the outbreak, the interim guidance WHO and some experts suggested that glucocorticoid treatment was not be used for COVID-19 due to insufficient clinical evidence 45, 46. However, subsequent studies revealed COVID-19 could benefit from that glucocorticoid treatment. One prospective meta-analysis of clinical trials showed that administration of corticosteroids could decrease 28-day all-cause mortality of critically ill patients with COVID-19 47. Another one controlled, open-label randomized trial reported preliminarily that dexamethasone led to lower 28-day mortality among those who were receiving either invasive mechanical ventilation or oxygen alone 48. Li recommended that glucocorticoid therapy could reduce the risk of 60-day mortality of patients who had a neutrophil-to-lymphocyte ratio (NLR) > 6.11 at admission 49. WHO changed glucocorticoid treatment guide that glucocorticoids are suitable for severe patients 50. In our study, the glucocorticoid treatment extended the survival time of the deaths of COVID-19 further supported the above conclusion.

There are an increasing number of applications of quantitative assessment with deep learning algorithms for COVID-19 lung lesions. Diagnosis and disease severity were assessed with the aid of artificial intelligence in COVID-19 patients 51, 52. Our model could accurately segment the lung tissue and quantitatively analyze the lesions on chest CT 14, 15. Li et al. used a deep convolution network to quantitatively evaluate COVID-19 lesions and analyze the disease severity using X-ray images 53. CRP, neutrophils, and procalcitonin were correlated with the volumes of pneumonia in patients who died from COVID-19, which indicated that these inflammation-related biomarkers were involved in the development of lung inflammation 54. Previous studies have identified that these biochemical indices were significantly correlated with an increased risk of death 55. This demonstrated the reliability of the quantitative model compared to other assessment methods. A deep learning model based on CT images provides a more convenient and fast way to assess risk factors for death apart from clinical text data. Combined quantitative CT imaging and clinical biochemical tests may be critical for improving the diagnostic efficiency of COVID-19.

Our study has some limitations. First, this study was retrospective, and a substantial amount of clinical information with dynamic changes could not be obtained. Prospective studies focusing on survivors and non-survivors may be of greater value. Second, many patients were admitted late in their illness due to medical resource shortages in the initial stage of the COVID-19. The collected data came from patients in different disease stages, which might have led to bias in the clinical information. Third, compared to the massive death tolls of COVID-19, the sample size of our study was limited, the results should be interpreted carefully. Multicenter studies with larger sample size are required. Fourth, this study is flawed in missing blood culture results although a large number of patients died for septic shock. Blood culture was not well performed mainly owing to practical reasons such as time-consuming. The best method to verify the incriminating microorganisms was still blood culture. It will help doctors choose the optimum antimicrobial treatment protocol.

## Conclusions

In summary, the majority of fatal patients with COVID-19 had more comorbidities such as hypertension, diabetes, and cardiovascular disease. The main causes of COVID-19 death were respiratory failure and multiple organ dysfunction syndrome. Respiratory failure, acute respiratory distress syndrome and septic shock were the most common serious complications. The use of antivirals, antibiotics, traditional Chinese medicines and glucocorticoids may prolong the survival time of deceased patients with COVID-19.

## Figures and Tables

**Table 1:** Demographic and clinical characteristics of recovered and deceased patients with COVID-19

	Total (n=2719)	Survivor (n=2610)	Non-survivor (n=109)	*P* value
Age, years	61.0 (50.0-68.0)	60.0(49.0-75.0)	73.0(66.0-81.0)	0.005*
**Sex**				
Male	1368 (50.2)	1297 (49.6)	71 (65.1)	0.001*
**Symptoms**				
Fever	1993 (73.3)	1918 (73.4)	75 (68.8)	0.280
Cough	1992 (73.3)	1921 (73.6)	71 (65.1)	0.050
Myalgia	790 (29.0)	762 (29.2)	28 (25.7)	0.429
Fatigue	1541 (56.7)	1477 (56.6)	64 (58.7)	0.661
Headache	125 (4.6)	121 (4.6)	4 (3.7)	0.637
Nausea	119 (4.4)	108 (4.1)	11 (10.1)	0.007*
Diarrhea	187 (6.9)	181 (6.9)	6 (5.5)	0.563
Sputum	477 (17.5)	460 (17.6)	17 (15.6)	0.585
Dyspnea	1083 (39.8)	1024 (39.2)	59 (54.1)	0.002*
Anorexia	1644 (60.5)	1583 (60.7)	61 (60.0)	0.327
**Comorbidities**				
Any	1402 (51.6)	1301 (49.8)	101 (92.7)	<0.001*
Hypertension	859 (31.6)	809 (31.0)	50 (45.9)	0.001*
Diabetes	399 (14.7)	368 (14.1)	31 (28.4)	<0.001*
Cardiovascular disease	211 (7.8)	180 (6.9)	31 (28.4)	<0.001*
Cerebrovascular disease	121 (4.5)	104 (4.0)	17 (15.6)	<0.001*
Chronic lung disease	103 (3.8)	92 (3.5)	11 (10.1)	0.002*
Chronic kidney disease	43 (1.6)	37 (1.4)	6 (5.5)	0.007*
Tumor	55 (2.0)	48 (1.8)	7 (6.4)	0.006*
Length from symptom onset to outcome, days	27.0 (19.0-37.0)	27.0(20.0-38.0)	23.5(16.8-34.2)	<0.001*
Length from hospital admission to outcome, days	13.0 (8.0-19.0)	13.0 (8.0-19.0)	9.0 (4.0-17.0)	<0.001*
**Cause of death**				
Respiratory failure	57 (2.1)	0 (0.0)	57 (52.3)	<0.001*
Multiple organ dysfunction syndrome	36 (1.3)	0 (0.0)	36 (33.0)	<0.001*
Septic shock	33 (1.2)	0 (0.0)	33 (30.3)	<0.001*
Sudden cardiac death	9 (0.3)	0 (0.0)	9 (8.3)	<0.001*
**Disease severity status**				
Mild/moderate	1915 (70.4)	1912 (73.3)	3 (2.8)	<0.001*
Severe/critical	804 (29.6)	698 (26.7)	106 (97.2)	<0.001*

Data are median (IQR), n (%), or n/N (%). Definition of abbreviation: COVID-19: coronavirus disease 2019; IQR: interquartile range. Outcome: death or discharge.

**Table 2:** Laboratory findings of recovered and deceased patients with COVID-19

	Total (n=2719)	Survivor (n=2610)	Non-survivor (n=109)	*P* value
White blood cells, ×10^9^/L	5.80 (4.71-7.12)	5.60 (4.71-6.82)	11.35 (8.38-14.01)	<0.001*
Neutrophils, ×10^9^/L	3.32 (2.58-4.69)	3.15 (2.48-4.22)	10.22 (7.33-13.44)	<0.001*
Lymphocytes, ×10^9^/L	1.53 (1.12-1.97)	1.58 (1.19-1.99)	0.52 (0.40-0.78)	<0.001*
Hemoglobin, g/L	120.0 (109.0-132.0)	121.0 (110.0-132.0)	110.0 (92.0-124.0)	0.090
Platelets, ×10^9^/L	201.0 (179.0-266.0)	224.0 (185.0-268.0)	132.0 (74.0-188.0)	<0.001*
Prothrombin time, s	12.2 (11.4-13.5)	12.0 (11.3-12.8)	15.5 (13.8-17.7)	<0.001*
Activated partial prothrombin time, s	31.0 (28.5-33.6)	31.1 (28.8-33.8)	33.6 (29.6-38.9)	0.578
D-dimer, mg/L	1.10 (0.87-1.69)	0.98 (0.62-1.57)	5.35 (2.05-9.43)	<0.001*
Albumin, g/L	36.80 (34.30-40.70)	38.50 (35.35-41.00)	29.45 (27.63-32.27)	<0.001*
Alanine aminotransferase, U/L	39.40 (36.50-43.20)	37.50 (32.20-41.30)	42.57 (21.66-76.36)	0.021*
Aspartate aminotransferase, U/L	37.80 (31.60-44.10)	35.60 (27.40-43.00)	52.18 (30.30-85.17)	0.009*
Urea, mmol/L	4.94 (4.08-6.21)	4.83 (4.02-5.86)	10.57 (7.30-15.54)	<0.001*
Creatinine, μmol/L	74.1 (65.3-88.1)	73.1 (64.5-85.5)	129.6 (75.7-212.9)	<0.001*
Creatine kinase, U/L	99.5 (82.5-125.0)	95.8 (75.0-104.4)	307.8 (91.2-901.7)	<0.001*
Glucose, mmol/L	5.41 (4.88-6.30)	5.31 (4.84-6.01)	8.89 (6.93-11.91)	<0.001*
C-reactive protein, mg/L	29.65 (24.03-38.65)	5.84 (3.04-17.74)	107.02 (63.63-144.83)	<0.001*
Procalcitonin, ng/mL	0.06 (0.04-0.13)	0.05 (0.03-0.08)	0.85 (0.25-3.63)	<0.001*
Interleukin-6, pg/mL	10.83 (9.39-17.93)	10.35 (9.20-14.99)	303.01 (37.82-1515.92)	<0.001*
Brain natriuretic peptide, pg/mL	120.30 (79.00-220.50)	125.10 (67.80-187.50)	238.56 (132.71-493.47)	<0.001*

Data are median (IQR), n (%), or n/N (%). Definition of abbreviation: COVID-19: coronavirus disease 2019; IQR: interquartile range.

**Table 3:** CT quantitative analysis of recovered and deceased patients with COVID-19

CT Finding	Total (n=2719)	Survivors (n=2610)	Non-survivor (n=109)	*P* value
GGO volumes, mm^3^	152662.1362 (367358.8235)	150449.881 (354389.0438)	432343.6163 (519041.9)	0.016*
Consolidation volumes, mm^3^	6548.5499 (31998.0062)	6285.4826 (30826.64013)	33617.26875 (129449.8)	0.059
Total lesions volumes, mm^3^	165649.804 (404778.3375)	159541.3268 (403494.9707)	544158.0301 (639793.4)	0.013*
Total lung volumes, mm^3^	4015204.6902 (1989081.5213)	4011129.176 (1944659.979)	3324305.205 (2546444)	0.690
The lesion ratio (%)	0.0407 (0.108)	0.0385 (0.10554)	0.13448 (0.22117)	0.013*

Definition of abbreviation: COVID-19: coronavirus disease 2019; GGO: ground-glass opacity; CT: computed tomography; IQR: interquartile range. The lesion ratio (%): total lesion volumes/total lung volumes.

**Table 4:** Complications and treatments of recovered and deceased patients with COVID-19

	Total (n=2719)	Survivors (n=2610)	Non-survivors (n=109)	*P* value
**Complications**				
Respiratory failure	131 (4.8)	68 (2.6)	63 (57.8)	<0.001*
Acute respiratory distress syndrome	63 (2.3)	34 (1.3)	29 (26.6)	<0.001*
Acute cardiac injury	89 (3.3)	72 (2.8)	17 (15.6)	<0.001*
Acute kidney injury	96 (3.5)	85 (3.3)	11 (10.1)	0.001*
Septic shock	71 (2.6)	31 (1.2)	40 (36.7)	<0.001*
**Treatments**				
Antibiotic treatment	1497 (55.1)	1401 (53.7)	96 (88.1)	<0.001*
Antiviral treatment	1720 (63.3)	1645 (63.0)	75 (68.8)	0.220
Glucocorticoid treatment	365 (13.4)	288 (11.0)	77 (70.6)	<0.001*
Traditional Chinese medicines treatment	2170 (79.8)	2088 (80.0)	82 (75.2)	0.224
Intravenous immunoglobin treatment	169 (6.2)	129 (4.9)	40 (36.6)	<0.001*
Targeted immunomodulatory treatment	102 (3.8)	85 (3.3)	17 (15.6)	<0.001*
COVID-19 convalescent plasma treatment	101 (3.7)	96 (3.7)	5 (4.6)	0.600
Oxygen therapy	1901 (70.0)	1819 (70.0)	82 (75.2)	0.217
Non-invasive mechanical ventilation	243 (8.9)	210 (8.0)	33 (30.3)	<0.001*
Invasive mechanical ventilation	166 (6.1)	98 (3.8)	68 (62.3)	<0.001*
Continuous renal replacement therapy (CRRT)	30 (1.1)	17 (0.7)	13 (11.9)	<0.001*
Extracorporeal membrane oxygenation (ECMO)	2 (0.7)	0 (0.0)	2 (1.8)	<0.001*

Data are median (IQR), n (%), or n/N (%). Definition of abbreviation: COVID-19: coronavirus disease 2019.

**Table 5:** Correlation between CT quantitative values and clinical parameters in recovered and deceased patients with COVID-19

	GGO volumes (mm^3^)		Consolidation volumes (mm^3^)		Total lesions volumes (mm^3^)		The lesion ratio (%)	
	Survivors	Non-survivors	Survivors	Non-survivors	Survivors	Non-survivors	Survivors	Non-survivors
Neutrophils	0.149*	0.336	0.162*	0.644*	0.155*	0.411	0.171**	0.575
CRP	0.276**	0.813*	0.293**	0.927**	0.285**	0.849*	0.267**	0.898*
PCT	0.117	0.206	0.082	0.763*	0.122	0.314	0.122	0.655
D-dimer	0.018	0.494	0.015	0.961**	0.013	0.602	0.057	0.912**
BNP	0.029	1.00	0.033	1.00	0.035	1.00	0.034	1.00

Abbreviations: COVID-19: coronavirus disease 2019; CRP: C-reactive protein; PCT: procalcitonin; BNP: brain natriuretic peptide; GGO: ground-glass opacity; CT: computed tomography. The lesion ratio (%): total lesion volumes/total lung volumes. *indicate P<0.05, **indicate *P*<0.01.
